# Environmental Determinants of Dengue Fever: ‎A Re‐Emerging Threat in the Middle East

**DOI:** 10.1002/hsr2.71177

**Published:** 2025-08-18

**Authors:** Farideh Bagherzadeh, Sara Hemati, Fazel Mohammadi‐Moghadam, Samira Sanami, Alizamen Salehifard, Sajjad Ahmad, Marzieh Farhadkhani

**Affiliations:** ^1^ Student Research Committee Shahrekord University of Medical Sciences Shahrekord Iran; ^2^ Department of Environmental Health Engineering, School of Health Shahrekord University of Medical Sciences Shahrekord Iran; ^3^ Abnormal Uterine Bleeding Research Center Semnan University of Medical Sciences Semnan Iran; ^4^ Department of Pediatrics, School of Medicine Hajar Hospital, Shahrekord University of Medical Sciences Shahrekord Iran; ^5^ Department of Health and Biological Sciences Abasyn University Peshawar Pakistan; ^6^ Educational Development Center Shahrekord University of Medical Sciences Shahrekord Iran

**Keywords:** aedes mosquitoes, dengue fever, environmental factors, Middle East

## Abstract

**Background:**

Re‐emerging of dengue fever (DF), a neglected disease‎, is a potential threat in tropical and subtropical countries. The increasing reporting of ‎positive ‎cases of DF in Middle East and consequently in Iran is a major reason for ‎concern. This paper ‎aims to describe the environmental aspect of DF to prevent a new crisis in Middle East.

**Methods:**

Literature search was conducted on PubMed, Web of Science, Scopus, Science Direct and Google Scholar and related data of each paper was evaluated.

**Results:**

Recent climate changes, increased rainfall, international travel, and tourism have produced ideal conditions for the ‎spread of Aedes mosquitoes‎ in Iran. In the absence of specific treatments and ‎vaccines, controlling mosquito populations is the most effective strategy to prevent dengue ‎transmission. This study ‎emphasizes the need for environmental monitoring and vector control to mitigate DF epidemics ‎in Iran. Also, collaboration between environmental and health professionals is needed to develop and implement effective strategies.

**Conclusion:**

These findings improve the understanding of the effects of the environmental factors on DF and support prevention and control strategies in the Middle East. Investing in research to understand environmental factors for mosquito surveillance and control are critical steps ‎towards sustainable and effective dengue management.

**Abbreviations**
DENV: dengue virus
DF: dengue fever
DHF: dengue hemorrhagic fever
EIP: external incubation period
ELISA: enzyme‐linked immunosorbent assay
FDA: Food and Drug Administration
GDP: gross domestic product
GIS: geographic information systems
IgG: immunoglobulin G
IgM: immunoglobulin M
NAAT: nucleic acid amplification tests
NS1: nonstructural 1
NSAIDs: nonsteroidal anti‐inflammatory drugs
PAHO: Pan American World Health Organization
WHO: World Health Organization

## 1 Introduction

Dengue fever (DF), a mosquito‐borne viral infection, poses a significant and growing public health challenge worldwide, particularly in tropical and subtropical regions [1, 2]. ‎ DF is caused by the dengue virus (DENV), which exists in four distinct serotypes (DENV‐1, DENV‐2, DENV‐3, and DENV‐4). The virus is primarily transmitted to ‎humans ‎through the ‎bites of infected female Aedes mosquitoes, especially *Aedes aegypti* and ‎*Aedes ‎albopictus* [3, 4]. ‎ The ‎clinical ‎presentation of DF ranges from asymptomatic infection to severe dengue ‎‎(also ‎known ‎as dengue hemorrhagic fever and dengue shock syndrome), which can be fatal if ‎not ‎managed ‎appropriately [5, 6]. ‎ Socioeconomic dynamics further compound the DF challenge in the Middle ‎East ‎and ‎Iran [2]. Many areas in these regions suffer from inadequate healthcare infrastructure ‎and ‎limited ‎access to medical services, which hampers timely diagnosis and effective ‎management of ‎DF [7, 8]. Additionally, there is often a lack of public awareness and ‎education about ‎dengue ‎prevention methods, which exacerbates the problem.‎ Robust environmental management is a basic, vital, and effective strategy to prevent and control the disease [7, 9]. *‎‎Aedes aegypti* first originated in Africa and spread to other continents through international travel. It has been identified in 129 countries worldwide. Approximately 50% of the world's population is currently susceptible to DF. It causes an estimated 40,000 deaths each year [4]. Although DF is traditionally not ‎considered endemic to Iran, recent reports suggest a ‎concerning ‎increase in DF cases ‎within the country [10]. The adaptability of Aedes ‎mosquitoes to various ecological niches has ‎facilitated their spread to ‎new territories, including ‎parts of Iran [7]. Recent ‎epidemiological data indicate a notable increase in DF ‎cases, ‎raising concerns about its ‎potential establishment and endemicity in Iran [11]. ‎Continuous monitoring of mosquito populations, viral activity, and ‎environmental ‎conditions can provide early warning signals for potential outbreaks, enabling timely and targeted interventions [12, 13]. The ‎environmental aspect of DF ‎transmission plays a pivotal role in ‎understanding the ‎dynamics of its spread and devising ‎effective prevention and control ‎strategies. Iran's unique ‎geographical and climatic conditions, ‎coupled with socioeconomic ‎factors, contribute to the ‎vulnerability of certain regions to DF outbreaks [10, 14]. Understanding the environmental ‎determinants of dengue transmission in Iran is ‎crucial for ‎developing targeted interventions and ‎mitigating the impact of this re‐emerging threat.‎

In light of the increasing threat posed by DF in Iran, this study aims to investigate the environmental determinants of dengue transmission and assess the effectiveness of existing control strategies. By identifying high‐risk areas and evaluating the ‎impact ‎of ‎environmental ‎factors on mosquito vector abundance and viral transmission ‎dynamics, ‎this ‎research seeks to ‎inform evidence‐based interventions for mitigating the burden of ‎DF ‎in Iran. By examining current trends ‎and ‎challenges, this study aims to offer ‎insights into ‎effective interventions and foster ‎collaboration ‎among public health officials, ‎researchers, and ‎communities to mitigate the impact ‎of DF in these regions.

## 2 Methodology‎

The literature search covered relevant studies published between 2000 and 2024. Databases including PubMed, Web of Science, Scopus, Science Direct, and Google Scholar were utilized. The search employed keywords such as “Dengue fever”, “Aedes mosquitoes”, “Environmental Factors” and “Middle East”. This review study focuses on recent research findings in DF prevention and control in Middle East. In addition, environmental factors that may have affecting the DF emergence were investigated.

## 3 Epidemic Scenario

The first written symptoms describing the possible occurrence of dengue were mentioned in 992 AD in a Chinese medical encyclopedia [15]. In 1906, scientists showed the transmission of diseases by the genus Aedes [8]. The first outbreak of dengue hemorrhagic fever (DHF) was first reported in 1953 in the Philippines. In 1981, the first outbreak of DHF occurred in this region of Cuba. This caused more than 300,000 cases of dengue, and 158 deaths, including 101 children [16, 17]. The first documented example of dengue causing a widespread epidemic was caused by serotype 1 in 1977. Which started in Jamaica, and spread to Cuba, Puerto Rico, and Venezuela [18]. The most likely time frame for the occurrence of a dengue epidemic is the period between 1779 and 1780 [19]. DF occurs in more than 100 countries across the Americas, Asia, Africa, and Australia, especially in tropical and subtropical regions of the world, and cases continue to increase worldwide. It also affects people who travel from countries where the disease is endemic. It is estimated that approximately 50 million infections occur each year [18, 20]. It is believed that the global epidemic of dengue began in Asia and the Pacific during and after World War II [21].

According to the report of the Pan American World Health Organization (PAHO), by mid‐May 2024, suspected cases of dengue in the Americas had reached more than 8.1 million, which is a 3.3‐fold increase compared to the same period last year. The countries with the most reported cases are Brazil, Argentina, Paraguay, Peru, Colombia, and Mexico. More than 3600 dengue deaths have been reported across the region. In February, Rio de Janeiro declared a public health emergency amid rising cases. Twenty regions of Peru are under health alert due to the outbreak of the epidemic, which has increased to more than 155,000 cases and killed 146 people [9]. Since 2008, DF has become a re‐emerging concern in Iran after the first case of imported DF was identified in a 61‐year‐old man with a history of travel to Malaysia [14]. Until August 30, 2024, that the DF began to spread in the Middle East, 221 cases have ‎been ‎confirmed in Iran and three deaths have been reported so far. Of these, 150 were imported (mainly from the United Arab Emirates) and 71 were locally transmitted, including 60 in Chabahar and 11 in Bandar Lengeh [22, 23].

## 4 Diagnosis

DF should be suspected in a patient who exhibits a sudden onset of fever, headache, body aches, and occasionally a rash that starts on the trunk. This patient should also reside in or have recently visited an area where the disease is common during the 2 weeks before the onset of symptoms [24]. The clinical presentation of DENV infection is influenced by factors such as the host's immunological status, age, and underlying medical condition. Relying just on clinical signs for diagnosing dengue is not dependable, as febrile sickness can present with a broad range of nonspecific symptoms. There are diagnostic instruments that are both specific and sensitive, and they are appropriate to use during specific stages of the disease [25]. Precise laboratory diagnosis of DENV infection is crucial, as any delay in detection heightens the likelihood of severe dengue and may result in an unfavorable disease outcome [26]. The diagnosis can be conducted by identifying the presence of the virus, viral nucleic acids, antigens, anti‐DENV antibodies, or a combination of these methods [24]. The Nonstructural 1 (NS1) antigen plays a crucial role in the replication of DENV within the host cell. The antigen is produced and released into the circulation of infected individuals, the high level of NS1 antigen in infected patient's sera makes it a crucial biomarker for early detection of flavivirus infection [27, 28]. Dengue can be diagnosed within the first 5 days of infection through virus isolation, RNA detection through nucleic acid amplification tests (NAAT), and reverse‐transcription PCR (RT‐PCR), as well as identifying antigens such as NS1 using immunoassays such as enzyme‐linked immunosorbent assay (ELISA). After more than 5 days since infection, it may no longer be possible to detect DENV RNA and antigens. This is because the viremia has decreased, and the body has started producing antibodies. At this time, the detection of anti‐DENV immunoglobulin M (IgM) and immunoglobulin G (IgG) produced in response to infection, using ELISA‐based serological assays, can help with the diagnosis of a DENV infection [29].

## 5 Treatment

DF does not currently have a specific therapy or cure. Current therapy options are supportive, with the goal of reducing complications and symptom severity. Paracetamol (acetaminophen) treatment alleviates symptoms such as muscle discomfort, fatigue, and fever [29]. Avoiding the use of nonsteroidal anti‐inflammatory drugs (NSAIDs) like ibuprofen and aspirin is advised due to their blood‐thinning and anticoagulant properties. This can exacerbate the prognosis of disorders that have a propensity for bleeding [29]. Fluid therapy is an essential component in the management of dengue. Oral fluid replacement is sufficient for treating DF, but severe cases require intravenous fluid replacement to prevent shock [30]. Dengvaxia (CYD‐TDV) was the first dengue vaccine approved by the United States Food and Drug Administration (FDA), and it was launched in Brazil in 2015 as a three‐dose series administered 6 months intervals for individuals aged 9–44 years [31]. In 2016, the World Health Organization (WHO) suggested using it in populations with a high disease burden, which means those with a seroprevalence of 70% or more [31]. In dengue‐endemic countries or regions, it is necessary to do screening before immunization to determine if the person has been previously infected with the DENV. The vaccine should only be administered to individuals who test positive. Currently, the limited usage of this vaccine is attributed to the requirement for pre‐vaccination screening [32]. CYD‐TDV is a live recombinant tetravalent dengue vaccine that has been developed by Sanofi Pasteur [33]. Qdenga (TAK‐003) is a newly developed attenuated tetravalent candidate that is currently showing promising outcomes. These results are based on a phase III study conducted on 20,000 children in Asia and Latin America (NCT02747927, estimated study completion August 2024) [34].

## 6 Result and Discussion

Over the past three decades, global concern about the public health effects of environmental pollution has increased. The WHO estimates that about a quarter of the diseases humans face today are due to long‐term exposure to environmental pollution [32]. Dengue transmission is affected by several factors, including climate changes and socio‐ecological factors. The Socio‐ecological factors associated with dengue incidence are gross domestic product (GDP), urbanization, travel, water bodies, and vegetation [35]. Climatic factors such as temperature, humidity, precipitation, wind speed, and air pressure can affect DF by influencing three essential environmental aspects, including DENV, mosquitoes, and transmission environment [36, 37].

### 6.1 Global Warming

Global warming has increased the likelihood of dengue transmission in previously nonnative locations, as these vectors can now thrive in environments that were previously unsuitable for them [38]. Temperature can affect both the physiology and behavior of vectors and the speed of virus replication. In the cold environment, the period of larval formation is longer, but higher temperature helps dengue viruses survive and increase their reproduction [39]. An increase in temperature can quicken the breeding of mosquitoes [40]. Higher temperatures are suitable regional threshold levels for the life cycle of dengue vectors, *Aedes aegypti* and *Aedes albopictus* mosquitoes. Temperature also shortens the external incubation period (EIP) and increases the frequency of mosquito bites [41]. In addition, the increase in temperature can increase the density of mosquitoes, and it also increases the flight distance of mosquitoes from 50 m below 18°C to 400 m (20°C–27°C) [42].

### 6.2 Climate Change

Infectious diseases sensitive to climate, under the influence of climate change and human population increase, pose an increasing threat to public health, so changes in climate patterns may affect the dynamics of their transmission [43]. DF is a climate‐sensitive disease. Climate plays an important role in the spread of dengue by affecting the life cycle of mosquitoes. Mosquitoes are known as ectotherms, meaning that their reproduction and activity are largely dependent on their surroundings [44]. Climate change is a critical worldwide challenge that may alter temperature and precipitation patterns, increase the risk of floods and droughts, and, in the long term, alter local hydrological conditions [45]. Changing natural environmental patterns related to the ocean‐atmosphere system can have direct and indirect effects on human health. The El Niño oscillation can lead to anomalies in the temperature and precipitation patterns of the world [46]. One of the biological consequences of the El Niño event is the spread of epidemics of diseases transmitted by insects, due to the decrease in rainfall and the increase in temperature in different regions of the world, which causes a high rate of mosquito reproduction [47].

In recent years, climate change in Iran and the Middle East has significantly affected the transmission patterns of DF. Studies show that the increase in temperature and decrease in rainfall caused by climate change have provided favorable conditions for the growth and reproduction of dengue‐carrying mosquitoes. For example, in the southern provinces of Iran, such as Hormozgan and Sistan and Baluchestan, warm and humid climatic conditions have increased the population of *Aedes aegypti* mosquitoes [48]. In addition, the El Niño phenomenon, by changing rainfall and temperature patterns, can lead to an increase in the prevalence of insect‐borne diseases, including dengue, in the region [49]. These factors, along with population growth and rapid urbanization in Iran, have contributed to an increased risk of dengue outbreaks in the country [50].

### 6.3 Rainfall Events

Stagnant water caused by rain plays an important role in mosquito breeding and growth because larvae live in water and their development takes about 7–10 days. Precipitation is an important element in climate models used to detect DF [51, 52]. The effect of rainfall on the occurrence of DF is applied through complex pathways, interacting with local topographical conditions, population, and vector species, other meteorological factors, and environmental parameters such as soil type and vegetation [6]. Rainfall events, in general, can provide breeding habitats for young Aedes mosquitoes and subsequently lead to increased mosquito abundance [53]. However, mosquito abundance and breeding habitats can be destroyed by heavy rains, which can dislodge larvae and pupae from breeding sites. Conversely, reduced rainfall can increase mosquito abundance as households increase the use of water storage containers [22, 54].

### 6.4 High Humidity

High humidity is associated with an increase in the incidence of DF [55]. The mosquito's respiratory system consists of an air tube in the form of a completely open spiral without any regulatory mechanism. When low humidity causes water to evaporate from the body, it causes body fluids to dry up [56]. Humidity affects mosquito lifespan, flight distance, reproduction rate, biting habits, and resting [57]. At a humidity of less than 60%, the lifespan of mosquitoes is shortened, and they are not carriers. At a humidity of 85%, the female mosquito reaches the age of 104 days, while the age of the male mosquito is 68 days. Newly laid eggs are sensitive to drying, and the survival rate of the adult mosquito is reduced due to the decrease in fertility [58].

### 6.5 Wind

**Figure 1:**
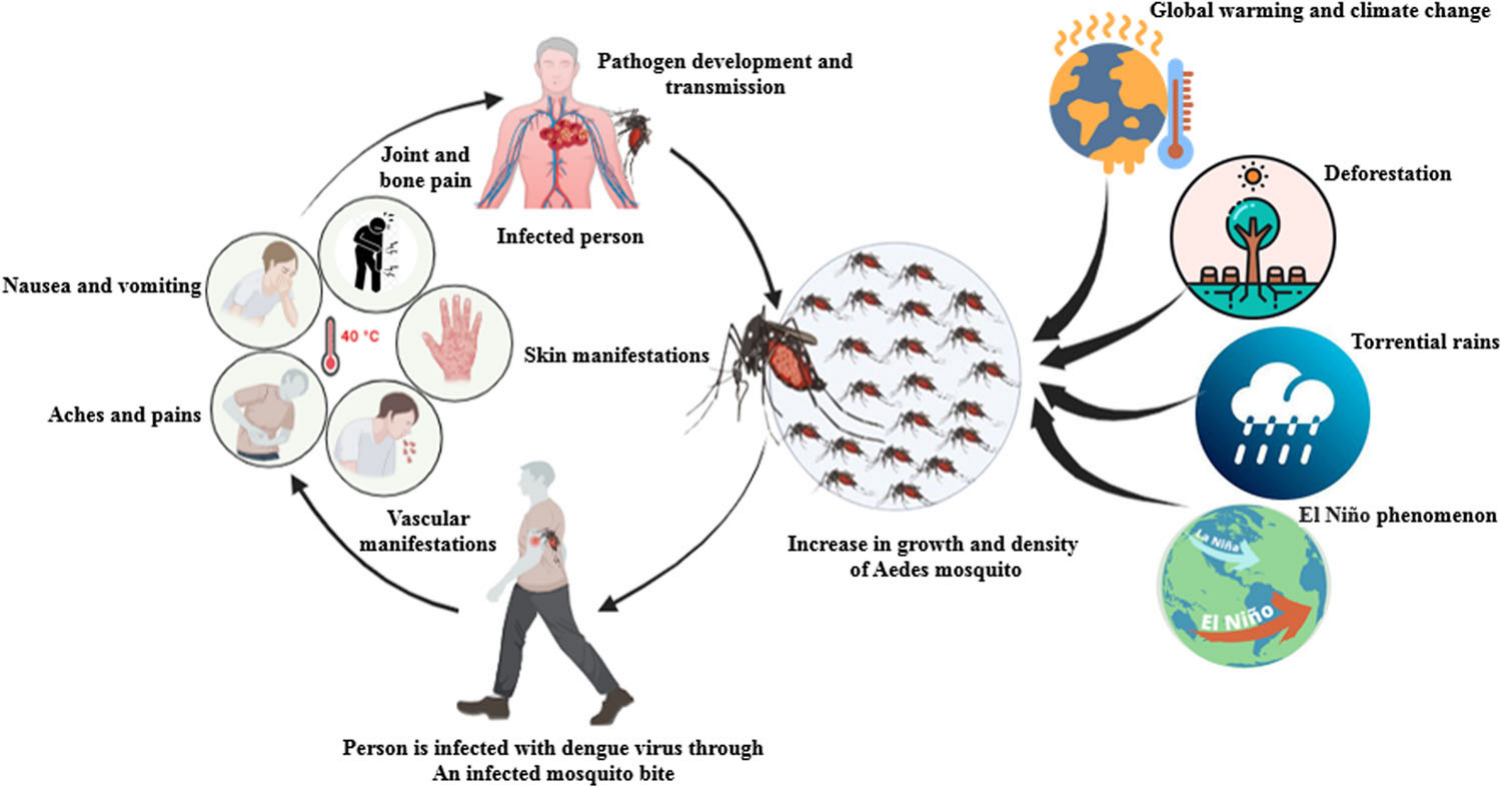
The factors affecting the Aedes mosquito transmission and DF diseases in humans.

Wind plays the least role in dengue infection. Adult mosquitoes typically do not survive the high wind speeds associated with hurricanes. On the other hand, stormy days are accompanied by humidity and rain, which increase the probability of mosquito reproduction. As a result, the risk of this disease increases steadily with increasing wind speed from 0 to 6 m/s [59]. The influencing factors on the Aedes mosquito transmission and DF spread are shown in Figure 1. This figure highlights the interaction between environmental stimuli, increasing vector mosquito populations, and clinical symptoms.

### 6.6 Socioeconomic and Urbanization Factors

Socioeconomic inequalities and patterns of urbanization play a key role in DF transmission. Rapid urban growth in the Middle East, including southern Iran, has led to increased population density, poor drainage systems, poor waste management, and erratic water supply—all of which create ideal conditions for Aedes mosquito breeding. In low‐income neighborhoods, the lack of piped water often necessitates water storage in open containers, which serve as prime mosquito habitats. In addition, limited access to healthcare, low public awareness, and weak disease surveillance systems hinder the timely detection and control of disease outbreaks. These factors, alongside climatic variables, significantly influence the regional dynamics of dengue transmission [13, 37, 60].

### 6.7 Limitations

Many studies have generally suggested a link between dengue fever and environmental factors, but this approach may lead to overgeneralization. Important factors such as differences in access to health services, level of education and public awareness, economic status, and health infrastructure also play a key role in the spread and control of the disease. Therefore, to fully understand the transmission of dengue fever in the Middle East, it is necessary to carefully examine the impact of socioeconomic and health factors in addition to environmental variables [50, 60]. Despite the breadth of the studies reviewed, many have limitations. Most studies have been limited to specific regions, limiting the generalizability of their findings to the entire Middle East region. Many studies also used small sample sizes, which reduces the statistical power of the results. The possibility of publication bias, especially in reports of positive results, is another challenge. Furthermore, comprehensive and long‐term epidemiological data are severely lacking to better understand the trend of dengue transmission in the region. A more detailed understanding of the socioeconomic factors influencing disease transmission also remains an important gap in the research literature that should be addressed in future studies.

## 7 Recommendations

### 7.1 Community Engagement

- Encourage cooperation between government agencies, nongovernmental organizations, and international groups to coordinate dengue control activities. Cooperation between government and nongovernmental organizations can effectively exchange resources, experiences, and knowledge. Effective responses to dengue outbreaks require coordination due to their widespread and dynamic nature.
- Launch large‐scale public health campaigns to educate communities about transmission, symptoms and preventive measures. Knowing the channels of virus transmission, early symptoms and preventive measures are vital to reduce the spread and severity of diseases.
- Dissemination of information content in social centers through social media. Providing materials through accessible venues and popular media channels will help reach a wider audience, especially in underserved areas, and strengthen community‐level preventive action.

### 7.2 Technology and Innovation

- Establish improved monitoring and surveillance systems for Aedes mosquito populations and dengue cases. Effective surveillance is very important for early detection of outbreaks and monitoring of the disease process. Mosquito population tracking and case reports enable timely action, reducing the impact of dengue in affected areas.
- Use GIS to identify and prioritize high‐risk areas. GIS technology helps identify and analyze high‐risk locations based on environmental and socioeconomic factors. This focused approach helps allocate resources efficiently to areas with the highest epidemic risk.
- Investigating new vector management methods, such as deploying drones for aerial monitoring of mosquito breeding sites. UAVs and other technologies can improve the efficiency of surveillance in hard‐to‐reach locations, provide valuable data about mosquito habitat, and enable targeted interventions to disrupt breeding sites.

### 7.3 Surveillance and Monitoring

- Carrying out regular and systematic vector control activities, such as eliminating stagnant water sources and using larvicides and adults. Regular removal of stagnant water and use of larvicides and adulticides reduce mosquito populations at different life stages, disrupting the dengue transmission cycle and reducing the risk of outbreaks.

Implementing environmental management techniques to limit breeding sites, especially in metropolitan areas. Urban areas are often ideal breeding grounds due to inadequate waste management and water management. By addressing these environmental factors, the risk of urban dengue can be significantly reduced and densely populated areas protected.

### 7.4 Intersectional Collaboration

- Include sectors such as environmental health, public works, and education in integrated dengue management strategies. A multi‐sectoral strategy for dengue control addresses various issues including waste management, infrastructure and public education to have a more sustainable and comprehensive impact on prevention.
- Encourage community participation in vector control measures, such as home and neighborhood cleanup programs. Community participation increases the effectiveness of vector control efforts. When people are involved in maintaining a clean environment and reducing breeding sites, mosquito populations can be better managed, and local transmission of dengue can be reduced.

## 8 Conclusion

In conclusion, understanding the environmental aspects of DF is paramount in addressing this re‐emerging threat in Iran. The intricate relationship between climatic conditions, urbanization, and mosquito breeding habitats underscores the importance of comprehensive environmental management strategies. Public health institutes must prioritize education and community engagement to foster awareness about the transmission and prevention of dengue. Healthcare providers need continuous training to recognize and manage dengue cases efficiently, while improved diagnostic facilities and the availability of necessary medical supplies are vital for effective treatment. Finally, investing in research to understand local environmental determinants and exploring innovative technologies for mosquito surveillance and control are critical steps towards sustainable and effective dengue management in Iran. By addressing these multifaceted challenges, Iran can mitigate the impact of DF and protect the community public health.

## Author Contributions

**Farideh Bagherzadeh:** conceptualization, methodology, investigation, validation, formal analysis, writing – original draft, writing – review and editing. **Sara Hemati:** conceptualization, methodology, investigation, validation, formal analysis, writing – original draft, writing – review and editing. **Fazel Mohammadi‐Moghadam:** methodology, investigation, writing – original draft, writing – review and editing. **Samira Sanami:** writing – original draft, writing – review and editing, investigation. **Alizamen Salehifard:** investigation, methodology, writing – original draft. **Sajjad Ahmad:** methodology, investigation, writing – original draft. **Marzieh Farhadkhani:** investigation, methodology, writing – original draft, conceptualization, writing – review and editing, validation, supervision.

## Conflicts of Interest

All authors have read and approved the final version of the manuscript, had full access to all of the data in this study, and take complete responsibility for the integrity of the data and the accuracy of the data analysis. The authors declare no conflicts of interest.

## Transparency Statement

The corresponding author, Marzieh Farhadkhani, affirms that this manuscript is an honest, accurate, and transparent account of the study being reported; that no important aspects of the study have been omitted; and that any discrepancies from the study as planned (and, if relevant, registered) have been explained.

## Data Availability

Data sharing is not applicable to this article as no new data were created or analyzed in this study.
